# Epidemiological and clinical features of human rabies cases in Bali 2008-2010

**DOI:** 10.1186/1471-2334-12-81

**Published:** 2012-04-02

**Authors:** Ni M Susilawathi, Agus E Darwinata, Ida BNP Dwija, Nyoman S Budayanti, Gusti AK Wirasandhi, Ketut Subrata, Ni K Susilarini, Raka AA Sudewi, Frank S Wignall, Gusti NK Mahardika

**Affiliations:** 1 Neurology Department, Faculty of Medicine Udayana University, Bali, Indonesia; 2 Microbiology Department, Faculty of Medicine Udayana University, Bali, Indonesia; 3 Sanglah Hospital, Denpasar, Bali, Indonesia; 4 Bali Provincial Health Office, Denpasar, Bali, Indonesia; 5 National Institute of Health Research and Development, Ministry of Health, Jakarta, Indonesia; 6 Oxford University Clinical Research Unit, Ho Chi Minh City, Vietnam; 7 Animal Biomedical and Molecular Biology Laboratory, Faculty of Veterinary Medicine Udayana University, Bali, Indonesia, Jl. Sesetan, Markissa 6, Denpasar, Bali 80225, Indonesia

**Keywords:** Rabies virus, Bali, RT-PCR

## Abstract

**Background:**

Previously thought to be rabies free, Bali experienced an outbreak of animal and human rabies cases in November 2008. We describe the epidemiological and clinical data of human rabies cases occurring in the first two years of the outbreak.

**Methods:**

We analysed the patient records of all rabies cases from the Sanglah General Hospital in Denpasar, and district hospitals in Buleleng and Tabanan. A conventional reverse transcriptase polymerase chain reaction was developed to detect the rabies virus genome in saliva, corneal swabs, and ante- and post-mortem cerebrospinal fluid (CSF).

**Results:**

There were 104 human rabies cases in Bali during November 2008-November 2010. Patients' mean age was 36.6 years (range 3-84 years; SD 20.7), most were male (56.7%), and originated from rural districts. Almost all (92%) cases had a history of dog bite. Only 5.8% had their wounds treated and received an anti-rabies vaccine (ARV) after the bite incident. No patients received rabies immunoglobulin (RIG). The estimated time from dog bite to the onset of signs and symptoms was 110.4 days (range 12-720 days; SD 118.2). The mean length of medical care until death was 21.8 hours (range 1-220 hours; SD 32.6). Less than 50% of patients had prodromal symptoms. The most frequent prodromal symptom was pain or paraesthesia at the bite site (37.6%). The two most common central nervous system infection signs were agitation (89.2%) and confusion (83.3%). Signs of autonomic nervous system dysfunction included hydrophobia (93.1%), hypersalivation (88.2%), and dyspnea (74.4%). On admission, 22 of 102 patients (21.6%) showed paralytic manifestations, while the rest (78.4%) showed furious rabies manifestations. The case-fatality rate was 100%. The rabies virus genome was detected in 50 of 101 patients (49.5%) with the highest detection rate from post-mortem CSF samples.

**Conclusions:**

Rabies is a major public health problem in Bali. Human fatalities occur because of a lack of knowledge regarding rabies risk, the poor management of dog bites, and the limited availability of RIG. Increasing public awareness of dog bite management, increasing the availability of ARV and RIG, and implementing an island-wide dog vaccination campaign will help prevent human rabies cases.

## Background

Rabies is a fatal neuropathogenic disease caused by the rabies virus, which is an enveloped RNA virus of the *Lyssavirus *genus, *Rhabdoviridae *family [1]. Rabies has a global distribution, with the exception of Antarctica, and infects domestic and wild animals. The rabies virus is transmitted to humans through the saliva of infected animals. The main route of infection is the bite of rabid dogs. Rabies is nearly always fatal when left untreated [2].

Rabies has been reported in Indonesia since the nineteenth century. The virus has been endemic in various islands surrounding Bali, including Sumatra, Java, Kalimantan, Sulawesi, and Flores since 2000 (review in [3]). Bali was considered rabies free until late November 2008, but an island-wide rabies outbreak has since occurred. Bali is a densely populated island with 3.9 million inhabitants in 5600 km^2 ^and with a dog-density of over 100 per km^2^, one of the highest in the world [4,5]. The number of dog bite incidents in Bali is high, with 21,806 reported in 2009 and 48,298 as of November 2010. The daily average of nearly one hundred dog bite incidents establishes the seriousness of the problem. Dog bites were common in Bali before 2008, but the number of victims seeking medical treatment was not high. Surveillance and recording of bite incidents was not conducted prior to 2008, because the risk of rabies was considered negligible. Rabies has been confirmed in both dogs and humans since November 2008 and 104 human cases were clinically diagnosed between November 2008 and November 2010. All human cases were fatal. We will now describe the epidemiological and clinical features of these human rabies cases occurring in Bali.

## Methods

### Data collection

Bali's health system includes Community Health Centres at the sub-district level, district hospitals, private hospitals, and one provincial referral hospital, namely the Sanglah General Hospital in Denpasar. After the outbreak of rabies began, some community health centres in the Badung and Tabanan districts were appointed as rabies treatment centres. Medical records were kept for all dog bite and human rabies cases seeking medical attention. The patient records from Sanglah and the district hospitals in Buleleng and Tabanan were the primary data sources of this study. Patients from other districts were referred to Sanglah Hospital for treatment. Data included the origin, age, and sex of the patient, estimated bite date, bite site, length of hospital care, presence of various prodromal clinical signs, and signs of central and autonomic nervous system dysfunction. We analysed all available data collected since the index human rabies case in November 2008.

### Ethical approval

Ethical clearance for this study was granted by the Research Ethics Committee of the Faculty of Medicine, Udayana University, Denpasar, Bali, Indonesia, reference number 723/Skrt/XI/2010 dated November 12^th^, 2010. Written informed consent was obtained from the patients' families before the samples and patient data were collected.

### Sample collection

Cerebrospinal fluid (CSF), saliva, and corneal swabs were obtained from the rabies patients. Ante-mortem CSF was collected by lumbar puncture. Post-mortem CSF was collected by sub-occipital puncture. Specimens were collected without preservative and stored directly at -80°C.

### Rabies genome detection and sequencing

Genomic RNA was isolated using a Pure-Link Viral RNA Isolation Kit (Invitrogen). Reverse transcriptase polymerase chain reaction (RT-PCR) was performed with a SuperScriptTM III One-Step RT-PCR System with Platinum^® ^*Taq *DNA Polymerase (Invitrogen) following the manufacturer's protocol. Primer set for the rabies RT-PCR was designed based on the rabies-virus sequence data from dogs in Indonesia that was available in GenBank using the web-based primer design tool Primer 3 http://biotools.umassmed.edu. The primer sequences used were 5'- TCAGGTGGTCTCYTTGAAGCC-3' (NF36) and 5'- ACGAACGGAAGTGGATGAAA-3' (NR836). A rabies-positive dog brain sample was used as positive control. This sample is available at the Animal Biomedical and Molecular Biology Laboratory of Udayana University. The sensitivity of the RT-PCR was 100% when compared with standard fluorescence antibody testing for rabies detection in dog brains (unpublished data). The RT-PCR conditions included incubation at 50°C for 60 minutes followed by incubation at 95°C for 7 minutes. The PCR cycle was 30 seconds at 94°C, 30 seconds at 55°C, and 60 seconds at 72°C. The cycle was repeated 35 times and followed by incubation at 72°C for five minutes. The RT-PCR was carried out using an MJMini Personal Thermal Cycler (Bio-Rad, Hercules, CA). The RT-PCR product was electrophoresed in 1% agarose gel stained with ethidium bromide and visualized in a UV transilluminator. For sequencing, the RT-PCR products were purified using a QIAquick PCR Purification Kit (Qiagen). Sequencing reactions were performed with a Big Dye Terminator v3.1 Cycle Sequencing Kit and run in an Applied Biosystem 3130/3130x Genetic Analyzer, which is an automated DNA sequencer. The sequencing work was performed at the Eijkman Institute for Molecular Biology, Jakarta, Indonesia. Trace identification was performed using Sequence Scanner Ver1.0 http://www.appliedbiosystems.com. The sequence was aligned using Mega4 software [6] and compared with the GenBank database using the basic local alignment search tool http://www.ncbi.nlm.nih.gov/blast/Blast.cgi.

## Results

**Figure 1:**
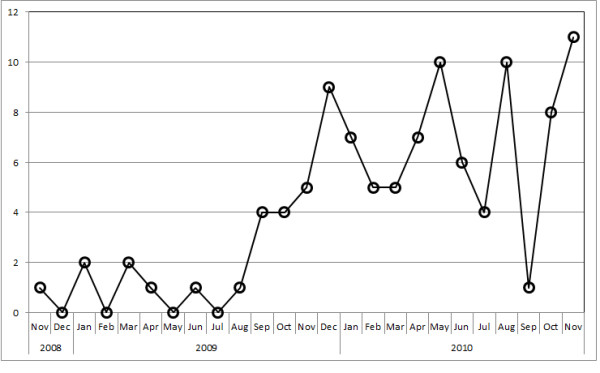
**Number of human rabies cases (persons) from November 2008 to November 2010 by month**.

**Figure 2:**
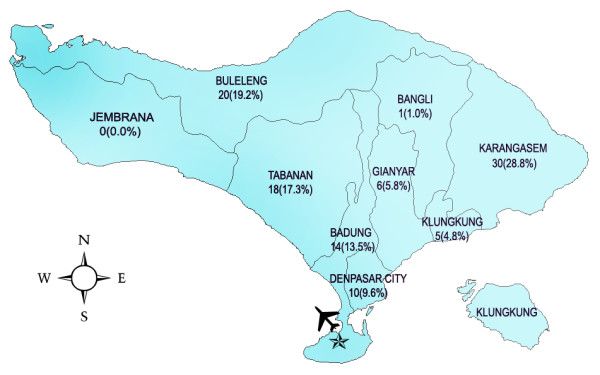
**Map of Bali Province and distribution of human rabies during November 2008-November 2010**. The districts and municipality described in the text are shown. The star symbol marks the location of the index cases of rabies in animals and humans. The aeroplane symbol is to show the Ngurah Rai International Airport as an orientation point.

Data of 104 patients, all of whom died of rabies in Bali from November 2008 until November 30, 2010, were used for analysis. The average patient age was 36.6 years (range 3-84 years, SD = 20.7). The victims were mostly male (56.7%). Figure 1 shows the number of human rabies cases by month for the initial two-year period of the outbreak. The increase in numbers of clinically diagnosed cases in 12 months after the initial case report can be seen. Cases were reported in eight districts (Figure 2) with most coming from rural districts, including Karangasem (28.8%), Buleleng (19.2%) and Tabanan (17.3%). The proportion from the Badung District, where the index case occurred was 13.5% (Figure 2).

Of the 104 cases, 96 had a history of dog bite. Dog-bite history information was missing for eight patients who were unconscious at presentation and whose family members were not aware of any incidents. Bites were most common in the lower extremities (59.3%), followed by the upper extremities (37.2%) and the head and neck (3.5%). Single bite cases were more frequent (72.9%) than multiple bite cases (27.1%).

Most patients (80.8%) had not undergone wound treatment of any kind, while 10.6% of them washed the wound themselves and 5.8% of them went to hospital on the day of the incident because of its severity, had their wounds cleaned and were given the anti-rabies vaccine (ARV). None of the cases received rabies immunoglobulin (RIG). Not all of the vaccinated patients completed their ARV regimens before developing the clinical symptoms of rabies.

The incubation period for the development of clinical rabies varied from as short as 12 days to as long as two years. In 98% of the cases, the incubation period was under one year. The incubation period was only 12-21 days when the bite site was on the head and neck, while it was 25 days to two years when on the extremities.

The list of clinical signs observed is presented in Table 1. Early recorded symptoms included pain or paraesthesia at the bite site (36.5%), nausea or vomiting (29.8%), fever (21.8%), myalgia (17.3%), headache (16.3%), and insomnia (6.7%).

**Table 1:** List of the clinical signs of the human rabies cases in Bali, November 2008-2010

Clinical Sign*	Number of Patient show the sign/total number of valid data	Frequency
Prodromal symptoms		
Pain/Paraesthesia at bite site	38/101	37.6%
Nausea and Vomiting	31/101	30.7%
Fever	22/101	21.8%
Myalgia	18/101	17.8%
Insomnia	7/101	6.9%
Headache	17/101	16.8%
Clinical sign of central nervous system infection		
Agitation	91/102	89.2%
Confusion	85/102	83.3%
Urine incontinence	28/102	27.5%
Flaccid Paralysis	22/102	21.6%
Seizure	16/102	15.7%
Hyperexcitability	14/102	13.7%
Abdominal discomfort	11/102	10.8%
Fasciculation and Tremor	4/102	3.9%
Cranial nerve involvement (Ophthalmoplegia, facial weakness and impaired swallowing)	3/102	2.9%
Autonomic dysfunction		
Hydrophobia	95/102	93.1%
Hypersalivation	90/102	88.2%
Aerophobia	76/102	74.5%
Dyspnea	76/102	74.5%
Photophobia	31/102	30.4%
Hyperhydrosis	27/102	26.5%
Piloerection	5/102	4.9%

*Clinical signs are ranked from the most to least common

The acute neurological signs were either furious (79.8%) or paralytic (20.2%). In the furious cases, 89.2% of patients showed intermittent agitation and confusion. Signs of autonomic dysfunction [7] were hydrophobia (93.1%), hypersalivation (88.2%), aerophobia (73.1%), dyspnea (74.5%), photophobia (29.8%) and piloerection (4.8%). Other signs included fever (18.2%), muscle fasciculation (3.8%), and convulsions (15.4%). Cranial nerve involvement and ophthalmoplegia, facial weakness and dysphagia were reported in 2.9% of patients. In the paralytic rabies cases, signs of flaccid paralysis were recorded in 21% of patients, urinary incontinence in 27.5%, and abdominal discomfort in 10.8%. The acute signs ended with abrupt death or progression into coma before death.

**Figure 3:**
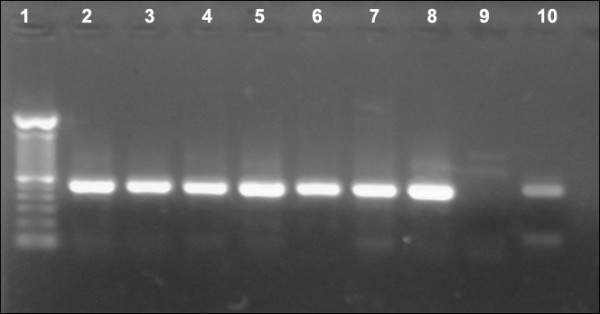
**Positive detection of rabies nucleoprotein from CSF samples following one-step RT-PCR**. Lane 1 was 100-bp DNA Ladder (Invitrogen). Lanes 1-8 were all positive CSF samples. Lane 9 was a rabies-negative dog brain as negative control. Lane 10 was a rabies-positive dog brain sample serving as a positive control.

Laboratory rabies diagnosis was carried with the RT-PCR technique on collected saliva, corneal swab, and ante- and post-mortem cerebrospinal fluid (CSF) specimens. A photograph of 1% agarose gel showing the positive detection of rabies nucleoprotein from CSF samples following one-step RT-PCR is presented in Figure 3. Rabies RNA was detected in 50 of 101 patients (49.5%). The highest detection rate was from post-mortem CSF specimens (detected in 8 of 15 patients). The RT-PCR detection rate from various specimens is presented in Table 2. The sequence of the rabies virus nucleoprotein from one patient has been deposited in GenBank with Accession Number JQ768453. This sequence showed a specific rabies genome fragment that was closely related to a rabies virus sequence from Indonesia that is available in GenBank.

**Table 2:** Summary of RT-PCR detection results from various specimens of human rabies cases in Bali 2008-2010

Specimen	Number of samples	Positives	Frequency of positive detection (%)
Saliva	101	28	27.7
Corneal Swab	86	4	4.6
Ante-mortem CSF	48	10	20.8
Post-mortem CSF	15	8	53.3

## Discussion

At the onset of the rabies outbreak, the human rabies management systems in Bali were unprepared. Doctors were not experienced in rabies diagnosis. Intensive training on rabies diagnosis and management was provided to health care workers following rabies confirmation in the index case. A case definition was developed under the guidance of the Ministry of Health [8], and it included clinical signs of rabies and a dog bite history. Initial case management often depended on the patient's complaints on arrival at the hospital and some misidentified cases ended up in cardiology for chest pain; others in internal medicine wards because of dyspepsia, typhoid fever, urinary colic, or paralytic ileus or pulmonology section for breathing difficulties, or abdominal surgery because of suspected appendicitis. Only after classical rabies symptoms appeared were patients transferred to Neurology departments for management. Given the largely passive rabies surveillance in Bali, it is possible that some cases will not have been recorded.

Standard diagnostic procedures were also not yet available in Bali at the onset of the rabies epidemic. Initial detection by RT-PCR was performed at Udayana University to confirm the outbreak. The initial goal was to find a positive sample that could be confirmed by sequencing, because RT-PCR sensitivity had not yet been evaluated. The standard fluorescent antibody test (FAT) [1] was not performed because the required equipment was not available. Samples collected included CSF, saliva, and corneal swabs along with ante- and post-mortem CSF. Cervical skin samples, although reported as a reliable source for diagnosis of human rabies [9], were not collected.

A diagnosis of rabies can also be established clinically and is the official standard for human cases [8]. Although the results of a RT-PCR test may be negative, rabies cases can still be clinically confirmed. A clinical diagnosis is not difficult if there is a history of a rabid or suspected dog bite. In other instances, rabies can be considered when there is an acute neurological disease that progresses to coma and death [1].

The Province of Bali, Indonesia, was not known to be a rabies endemic area prior to November 2008. The index human rabies case was reported in the Badung District (Figure 2) and it was followed by rabies detection in many dogs. The local government control policy was initially a combination of dog vaccination and dog elimination. However, the movement of dogs during the incubation period to rabies-free areas probably contributed to the spread of the virus (Figure 2). By early 2010, the disease had spread throughout the entire island and the initial extensive dog culling was not successful in stopping the outbreak. Data also indicated that most cases occurred in the Karangasem and Buleleng districts in densely populated but rural areas. Large numbers of semi-feral, unvaccinated dogs along with a lack of rabies knowledge, and poor socioeconomic conditions common to other outbreaks [10,11] also contributed to uncontrolled rabies spread among dogs and the poor management of human infections.

This report illustrates that most patients did not receive proper first aid, rabies vaccination, or passive immunization post-exposure. Of 104 patients, only 5.8% sought medical help and received post-exposure vaccination. RIG was not available to them. Among those given initial treatment, the vaccination regimens were not completed because of a short incubation period and the onset of symptoms within two weeks of bites on the head and neck region.

All epidemiological features of the human rabies cases in Bali resemble those reported elsewhere. Rabies victims in Bali were mostly male, similar to China [11]. The bite locations were mostly on patients' legs and arms. Bites on the head and neck were less frequent. In China [11] and Tanzania [12], bites on the head and neck contributed significantly to the number of human rabies cases. The age distribution of rabies patients in Bali was similar to those in reported in China and Taiwan.

Dogs were the source of human rabies infections in Bali. Infections in other animals that might theoretically transmit the virus to humans, such as cats and bats, have not yet been confirmed. Dogs are known as the most common source of rabies transmission to humans throughout the world, especially in Asia, Latin America, and Africa [1].

In this study, the incubation period of human rabies ranged from 60-90 days post bite in most cases. A shorter incubation period of 12-21 days was recorded in patients with bites around the head and neck. This is a typical picture of rabies in humans. It is well understood that the rabies incubation period is one of the most variable among all infections. The average incubation period is around 1-3 months but may range from less than 7 days to 6 years [1,13]. The incubation period depends on several factors such as bite site, virus quantity in the saliva, type and depth of the bite wound, and viral virulence [14]. Longer incubation periods might lead to problems in making a diagnosis when the history of a bite might be forgotten [15].

Rabies infection in humans is triphasic, i.e., prodromal, acute neurologic, and coma proceeding to death [16]. The prodromal signs are mostly non-specific, including fever, headache, myalgia, nausea, vomiting, and abnormal sensation around bite sites, such as itching, burning, numbness, or paraesthesia [17]. These non-specific complaints may lead to a misdiagnosis of rabies at this early stage [15]. In nearly 50% of paralytic rabies cases and 30% of furious rabies cases, local manifestation in the form of itching, pain or paresthesia at the bite site may be the earliest symptom [13].

The vagueness of prodromal signs was evident in the human rabies cases in Bali. The three most common prodromal signs observed in this study were paraesthesia at the bite site, nausea, vomiting, and fever. In Bali, about 30% of the rabies patients complained of these signs on admission to the medical facilities.

Most patients (98%) were in the acute neurological phase on arrival at the hospitals. The most dominant signs were acute neurological and autonomic dysfunction. Typically, human rabies patients show anxiety, agitation, dysphagia, hypersalivation, paralysis, and episodes of delirium. A case is classified as being furious rabies if the hyperactivity signs are dominant, or as paralytic or dumb rabies if the patient presents with varying degrees of paralysis and lethargy (review in [1]). However, this classification does not seem to be clear-cut. Clinically, furious rabies should be easier to establish than paralytic rabies. The paralytic type is caused by virus infection in the spinal cord [18]. In some cases of human rabies in Bali, the patients showed paralytic symptoms on admission and then developed furious symptoms in the later stages.

The standard methods for rabies diagnosis in humans are virus isolation, antigen detection, and viral genome detection [19-21]. Confirmation should be made after virus detection using FAT and immunohistochemistry from CNS specimens [22,20,23]. The infrastructure to perform such testing was not available in Bali. RT-PCR has been recommended to detect virus specific RNA [20-24]. The specimens selected for this technique include brain, saliva, and other affected tissues. Accordingly, a RT-PCR system was developed to detect and confirm human rabies infections in Bali. Primer sets were selected based on an existing Indonesian rabies-genome database available in GenBank, and commercially purchased. The system was proven to detect the rabies genome in patients. RT-PCR has been reported to have a high specificity (up to 100%) and a moderate level of sensitivity [13]. This is similar to our detection level of about 50% of the clinical rabies patients. Rabies infection cannot be ruled out of the RT-PCR negative specimens. Our observations indicate that among the samples taken, the best sample for RT-PCR detection is CSF. Other publications found a higher PCR positivity rate early in the disease progression when neutralizing antibodies are not present or are at a low titre [13]. While a positive PCR result is indicative of rabies, a negative result does not exclude it.

Rabies prevention is all about awareness. The public must be informed about all relevant aspects of rabies and rabies control. Community initiatives should ensure sustainable control of canine rabies and proper management of dog bites to prevent rabies infections in humans. Such initiatives rely on community knowledge and a willingness to cooperate. Information on the cause of rabies, its seriousness, how it is transmitted, its signs in animals and humans, and on the medical management of dog bites need to be widely disseminated. The adequacy of government policies on rabies control should be reviewed and international policy standards should be met. The most efficient measure for human protection is actually the vaccination of dogs. As proven worldwide, the elimination of canine rabies and the prevention of human rabies mortality is feasible through mass vaccination of domestic dogs [12,25-27]. Public awareness must be continuously maintained, not only in rabies infected areas but also on neighbouring islands that have never experienced the disease [28,29].

Unsuccessful control of canine ra cbies and inadequate post-exposure prophylaxis (PEP) in humans are the main factors leading to the high incidence of human rabies infection in Bali. Publicity and education about the risk and prevention of rabies, as well as high rates of dog vaccination, properly trained medical personnel, and adequate vaccine and RIG supplies are necessary and important for the elimination of human rabies cases and the care of those potentially exposed.

## Conclusions

Rabies has emerged as a major public health problem in Bali. Human rabies fatalities have occurred because of a lack of knowledge regarding rabies risk and poor management of dog bite incidents, including poor wound cleaning and failure to seek PEP. The limited availability of RIG for the treatment of high-risk rabid dog bites also contributed to the high fatality rate. Public awareness regarding dog and wound management, as well as ensuring the availability of ARV and RIG are the keys to preventing human rabies cases. An island-wide vaccination campaign should be undertaken to eliminate canine rabies.

## Competing interests

The authors declare that they have no competing interests.

## Authors' contributions

Ni M Susilawathi, Gusti A K Wirasandhi, and Ketut Subrata contributed to data collection at the provincial and district hospitals. Agus E Darwinata, Ida B N P Dwija, and Nyoman S Budayanti contributed to rabies genome detection, data entry, and data analysis. Ni K Susilarini contributed to obtaining ethical clearance. Frank S Wignall contributed to project planning and supervision. Raka AA Sudewi and Gusti NK Mahardika planned and co-supervised the study and drafted the manuscript.

## Pre-publication history

The pre-publication history for this paper can be accessed here:

http://www.biomedcentral.com/1471-2334/12/81/prepub
